# Age-induced changes in lung microenvironment: from melanoma dormancy to outgrowth

**DOI:** 10.1038/s41392-022-01303-5

**Published:** 2023-01-16

**Authors:** Juan Jin, Li Li, Caiyun Fu

**Affiliations:** Zhejiang Provincial Key Laboratory of Silkworm Bioreactor and Biomedicine, College of Life Sciences and Medicine, Zhejiang Sci-Tech University, 310018 Hangzhou, China

**Keywords:** Cancer microenvironment, Skin cancer

**Fig. 1:**
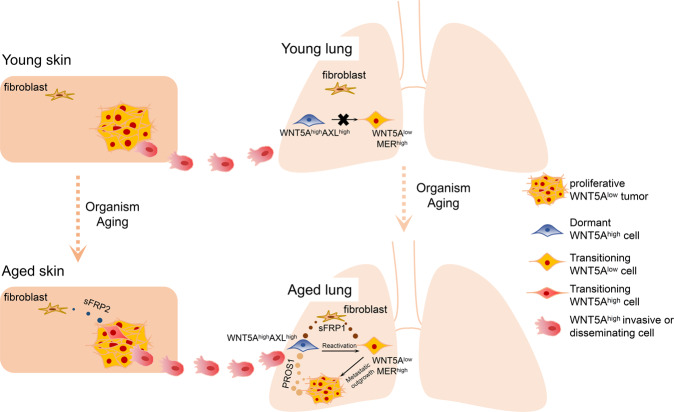
Age-induced changes promote metastatic melanoma outgrowth. Aged skin fibroblasts promote melanoma cell dissemination from the primary tumor through sFRP2 secretion which activates WNT5A. Aged lung fibroblasts secrete sFRP1 to reduce WNT5A expression of disseminated melanoma cells, thus promoting metastatic melanoma outgrowth from dormancy through AXL-MER dormancy-reactivation axis

In a recent study published in *Nature*, Mitchell E. Fane et al. revealed the critical role of the aged microenvironment in the reactivation of melanoma cells from dormancy.^1^ The authors found that age-induced lung fibroblast secretory changes promoted growth activation of dormant melanoma cells in the lung, and age-induced skin microenvironment changes suppressed melanoma growth but promoted melanoma cell dissemination (Fig. 1).

Cancer cells from primary tumors can disseminate and seed in distant tissues but may remain dormant and form overt metastases after several years.^2^ This may explain why tumor metastasis may occur even many years after curative therapeutic approaches. However, the underlying mechanism of tumor dormancy is poorly understood. In melanoma, older patients have a poor prognosis and a high risk of recurrence.^3^ It was previously shown that melanoma cells intradermally implanted into aged mice formed larger lung metastases than those implanted in young mice.^4^ The differences in lung metastasis due to the increase of dissemination from aged primary tumors or the changes in the aged lung microenvironment needed to be further explored.

In this study, the researchers implanted melanoma cells in the skin of young or aged mice and found that the primary tumor grew more slowly in the aged mice, while larger metastatic colonies formed in the aged lung. Although single melanoma cells were seeded in the lung in similar numbers, the metastasis was more effective in aged mice. Then, the researchers co-cultured melanoma cells with human healthy skin or lung fibroblasts and found that melanoma cells in the aged lung fibroblast microenvironment proliferated faster than those in the young in vitro. Conversely, melanoma cells in the aged skin fibroblast microenvironment proliferated slower than those in the young skin fibroblast microenvironment. These data suggested that changes in secreted soluble factors promoted these phenotypic differences. To test this hypothesis, a proteomic analysis of the secretome of the lung fibroblasts from healthy young and aged individuals was performed. The results showed that aged lung fibroblasts secreted more sFRP1, a non-canonical WNT antagonist. WNT5A was shown to promote prostate cancer cell dormancy in bone.^5^ Thus, the researchers hypothesized that sFRP1 may reactivate dormant melanoma cells and resume proliferation by inhibiting WNT5A signaling. To confirm this hypothesis, they treated melanoma cells with conditioned medium from lung fibroblasts and found that treatment with conditioned medium from aged lung fibroblasts significantly inhibited non-canonical WNT5A signaling in melanoma cells and reduced dormancy-related gene expression, whereas increased the expression of proliferation-related genes. Then, the researchers treated melanoma cells with recombinant sFRP1 and found that it could also inhibit WNT5A signaling, reduce the expression of dormant marker genes and increase the expression of proliferation markers. To confirm these data in vivo, the researchers injected an sFRP1-neutralizing antibody into mice after inoculating melanoma cells intradermally in aged mice. The results showed that the number of metastatic colonies in the lungs of mice after the neutralization of sFRP1 was significantly reduced. These data suggest that the aged lung fibroblasts can reactivate melanoma cell dormancy by secreting sFRP1, thereby promoting metastatic tumor formation.

In a previous study, the researchers demonstrated that aged skin fibroblasts secreted sFRP2, which promoted the WNT5A signaling pathway, thus inducing a slow-cycling but invasion melanoma phenotype.^4^ They found that sFRP2 promoted cell invasion and rendered melanoma cells more sensitive to oxidative stress by downregulation of β-catenin. To further explore the role of WNT5A in the dissemination and dormancy of melanoma cells, they used Dox-inducible knockdown to reduce the expression of WNT5A starting at day 3 or day 21 after intradermal melanoma cell implantation. They found inducible knockdown at either time point promoted primary tumor growth. However, only mice treated with Dox starting at day 21 formed larger lung metastatic colonies. It was noteworthy that fewer single disseminated melanoma cells seeded in the lung of mice with inducible knockdown beginning at day 3. Overall, these data show that temporal downregulation of WNT5A promotes the reactivation of dormant cancer cells, and allows metastatic outgrowth even in previously growth-restrictive young mouse lungs.

TYRO3, AXL, and MER receptors (TAMs) are three tyrosine kinase receptors. In melanoma, studies have shown that AXL expression was positively correlated with WNT5A. MER and TYRO3 had an inverse expression pattern to that of AXL. They hypothesized that AXL versus either MER or TYRO3 may form a dormancy-reactivation axis during metastatic melanoma. The researchers found that downregulation of MER was required for efficient dissemination from the primary tumors and increasing MER promoted the reactivation of disseminated melanoma cells from dormancy in the lung. AXL overexpression in melanoma cells reduced the expression of MER and decreased metastatic colonies with unchanged WNT5A, implying that AXL is downstream of WNT5A. PROS1, which was the prominent ligand for MER, was expressed higher in young metastases. Of note, MER^high^ melanoma cells secreted more PROS1 and PROS1 treatment induced larger colonies in the young mouse lung.

Taken together, Mitchell E. Fane et al. demonstrated the complexity of the WNT signaling pathway in melanoma cell dormancy and metastasis, which was modulated by age-induced changes in fibroblasts. They also identified a dormancy-reactivation axis in which AXL-MER converges downstream of the WNT5A signaling pathway to reactivate dormant tumor cells. That may explain why elderly patients with melanoma have a high risk of tumor metastasis and recurrence. The aged tumor microenvironment, which is involved in tumor metastasis, should be a potential target for tumor therapy. And age should be considered as a parameter in the design of cancer treatment strategy. However, this study specifically focused on age-induced changes in the lung only. Further study still needs to explore the metastatic microenvironment, owing to the fact that melanoma usually metastasizes to other distal sites, including the brain and liver. In a conclusion, these findings have important implications for the understanding of tumor dormancy and metastasis, thus providing new directions for designing treatment strategies for metastatic tumors.
